# High mirror symmetry in mouse exploratory behavior

**DOI:** 10.3389/fnbeh.2024.1381852

**Published:** 2024-04-29

**Authors:** Ehud Fonio, Ofer Feinerman

**Affiliations:** Department of Physics of Complex Systems, Weizmann Institute of Science, Rehovot, Israel

**Keywords:** animal behavior, operational space, constraints, exploration, symmetry, mouse, locomotion, memory

## Abstract

The physicality of the world in which the animal acts—its anatomical structure, physiology, perception, emotional states, and cognitive capabilities—determines the boundaries of the behavioral space within which the animal can operate. Behavior, therefore, can be considered as the subspace that remains after secluding all actions that are not available to the animal due to constraints. The very signature of being a certain creature is reflected in these limitations that shape its behavior. A major goal of ethology is to expose those constraints that carve the intricate structure of animal behavior and reveal both uniqueness and commonalities between animals within and across taxa. Exploratory behavior in an empty arena seems to be stochastic; nevertheless, it does not mean that the moving animal is a random walker. In this study, we present how, by adding constraints to the animal’s locomotion, one can gradually retain the ‘mousiness’ that characterizes the behaving mouse. We then introduce a novel phenomenon of high mirror symmetry along the locomotion of mice, which highlights another constraint that further compresses the complex nature of exploratory behavior in these animals. We link these findings to a known neural mechanism that could explain this phenomenon. Finally, we suggest our novel finding and derived methods to be used in the search for commonalities in the motion trajectories of various organisms across taxa.

## 1 Introduction

In his recent preprint study: “On growth and form of animal behavior” (Golani, 2022), distilling a lifetime of scientific work, Ilan Golani admirably aspires to bridge a long-standing gap between the diverse modern behavioral research and the classical ethological study. Although modern behavioral research, which ultimately seeks to decipher the intimate recurrent relation between genes, neurology, and environment and the behavior to which it relates, has greatly broadened the accessibility to measuring behavior, several deep intuitions and principles from classical ethology have been neglected. This unfortunately has been delaying the expected development in the study of animal behavior, especially compared to the revolutionary progress in the other related research fields mentioned above. By integrating his ideas and findings from his long research, Golani discloses a unifying theory of ethology for the study of motion-based behavior. One of the principles, taken from the tradition of comparative anatomy, is the understanding that behavior is the extension of anatomy [see Golani (1992)]. An implication of this insight, says Golani, is that the connectedness of the skeleton, which implies specific mechanical constraints, is mirrored in movement (Golani, 2022). The subject of behavioral constraints that are manifested by the reduction of the animals’ operational space frequently appears throughout Golani’s research (Golani et al., 1979, 1981, 1997; Golani and Moran, 1983; Golani, 1992; Tchernichovski et al., 1996; Gakamsky et al., 2013; Gomez-Marin et al., 2016; Golani, 2022).

We would like to go further and widen this angle by using an apophatic approach that originated in philosophy and applied in theological studies (Putnam, 1997) as well as in modern science (Bridewell and Isaac, 2021). We show how the behavior of a certain animal can be considered as the multidimensional subspace that remains after secluding actions that are not available to the animal or even statistically less likely to occur due to constraints. Whether it is the physicality of the world in which the animal operates, its anatomical structure, physiology, or its perception, emotional states, and cognitive capabilities, all determine the dynamic boundaries of the organism’s freedom of operational space. We argue that understanding animal behavior can be achieved not just by the positive description of its characteristics but also negatively by characterizing the behavioral boundaries from “outside,” exposing the constraints and limitations that carve the intricate structure (geometry) of movement-based behavior, thus revealing both uniqueness and commonalities between animals at the ontogeny as well as phylogeny levels.

In a previous study, we allowed mice to freely explore a novel area from the safety of their home cage. This led to the discovery of a gradual build-up in the extent and amplitude of the degrees of freedom that became available to the Balb/c mice and was reflected in the shift from highly predictable behavior to seemingly stochastic movement (Fonio et al., 2009). These findings revealed a highly preserved sequence of building blocks and an incremental addition of dimensions that constitutes the morphogenesis of exploratory behavior starting from ordered and restricted motion, which increasingly becomes more unpredictable as the animals become more familiar with the novel environment. These results coincide with earlier findings about a similar build-up in the animal’s egocentric perspective (Golani, 2012). Nevertheless, the increase in the freedom of movement does not mean that the animal becomes a random walker. We argue that the very essence of being a certain animal is reflected by the signature of the various constraints that shape its behavior, provide its unique point of view—umwelt (von Uexküll, 1934)—and define its preferences and affordances (Gibson, 1979). Following this line of thought, “mousiness” is the body of behavioral space that is allowed within all constraints, either physical, cognitive, or emotional, that limits the much wider possible motion space. The home-base phenomenon, for example, where the behavior is organized concerning an allocentric point of reference, which increases the predictability of the behavior (Eilam and Golani, 1989), nicely represents this principle, allowing both compression and segmentation of the behavior to be meaningful building blocks.

In this study, we start with a somewhat simplified demonstration of how adding constraints can carve the behavioral space of the exploring mouse, gradually regaining its unique “mousiness.” Then, we present an intriguing high mirror symmetry along the animals’ trajectory during exploratory behavior, highlighting a novel constraint that compresses the complex nature of exploratory behavior in Balb/c mice. We discuss a possible link of the high mirror symmetry findings to a known mechanism of reversed replay of neural activity that could explain this phenomenon. Finally, we discuss the benefits of the complementary approach of finding constraints as a beneficial method for behavioral descriptions that could reveal homologies across taxa and a much-needed source for well-defined patterns in interdisciplinary research fields that involve genetics and neurological studies.

## 2 Methods

### 2.1 Animals and housing

Twelve male BALB/c mice (11 weeks of age, Harlan Laboratories) were kept in a 12:12-h light cycle (Light: 6:00 AM to 6:00 PM), singly housed due to their known inter-male aggressiveness for 2 weeks before testing, at 22°C room temperature with water and food *ad libitum*, and maintained in facilities fully accredited by the National Institutes of Health (NIH) Animal Welfare Assurance Number A5010–01 (TAU). The studies were conducted following the Guide for Care and Use of Laboratory Animals provided by the NIH, “Principles of Laboratory Animal Care” (NIH publication no. 86–23, 1996).

### 2.2 Experimental design and data acquisition

The experimental setup consisted of a large 250-cm-diameter circular arena, having a non-porous gray floor, illuminated with an IR projector (880-nm) and dim white light (<1 Lux) that was placed on the ceiling above the arena center, simulating moonlight. The arena was confined by a 60-cm-high continuous wall with a single 4 × 5 cm doorway leading to an infra-red lit Plexiglas home cage (30 × 40 × 50 cm) containing wood shavings from the original home cage, food, and water *ad lib*. The home cage was firmly attached to the outer side of the arena, securing a free passage between the arena and the home cage interior (no corridor). Curtains from the ceiling to the floor surrounded the arena and separated it from the rest of the experimental room. Animals were recorded by a DVR system at 25 fps and spatial resolution of 1 pixel = 1 cm and were tracked live using Noldus EthoVision (Spink et al., 2001). The arena was thoroughly rinsed with water and soap and then dried, and the home cage was replaced by a clean home cage at the end of each mouse session.

### 2.3 Behavioral testing protocol and data processing

Each mouse was housed in the home cage for a 24-h adjustment period before the experiment initiation. To increase the likelihood that the mouse’s activity was elicited by the exposure to the novel open space rather than by the diurnal cycle, the session commenced 4 h after the onset of the light cycle, which is the non-active part of the cycle of mice, at 10:00 am. At this time, the doorway barrier that was blocking the passage between the home cage and the arena was gently removed. This door was left open throughout the session. The infrared and dim lights were switched on when the mouse was introduced into the home cage (24-h before door removal). The BALB/c mice session was extended over 45 h. Data processing and analysis of the mouse trajectories included smoothing (Hen et al., 2004) and segmentation (Golani et al., 1993) using SEE, a software-based strategy for exploring exploration (Drai and Golani, 2001). All the above information is taken from the publication of the original research (Fonio et al., 2009).

### 2.4 Reconstructing “mousiness”

**Figure 1:**
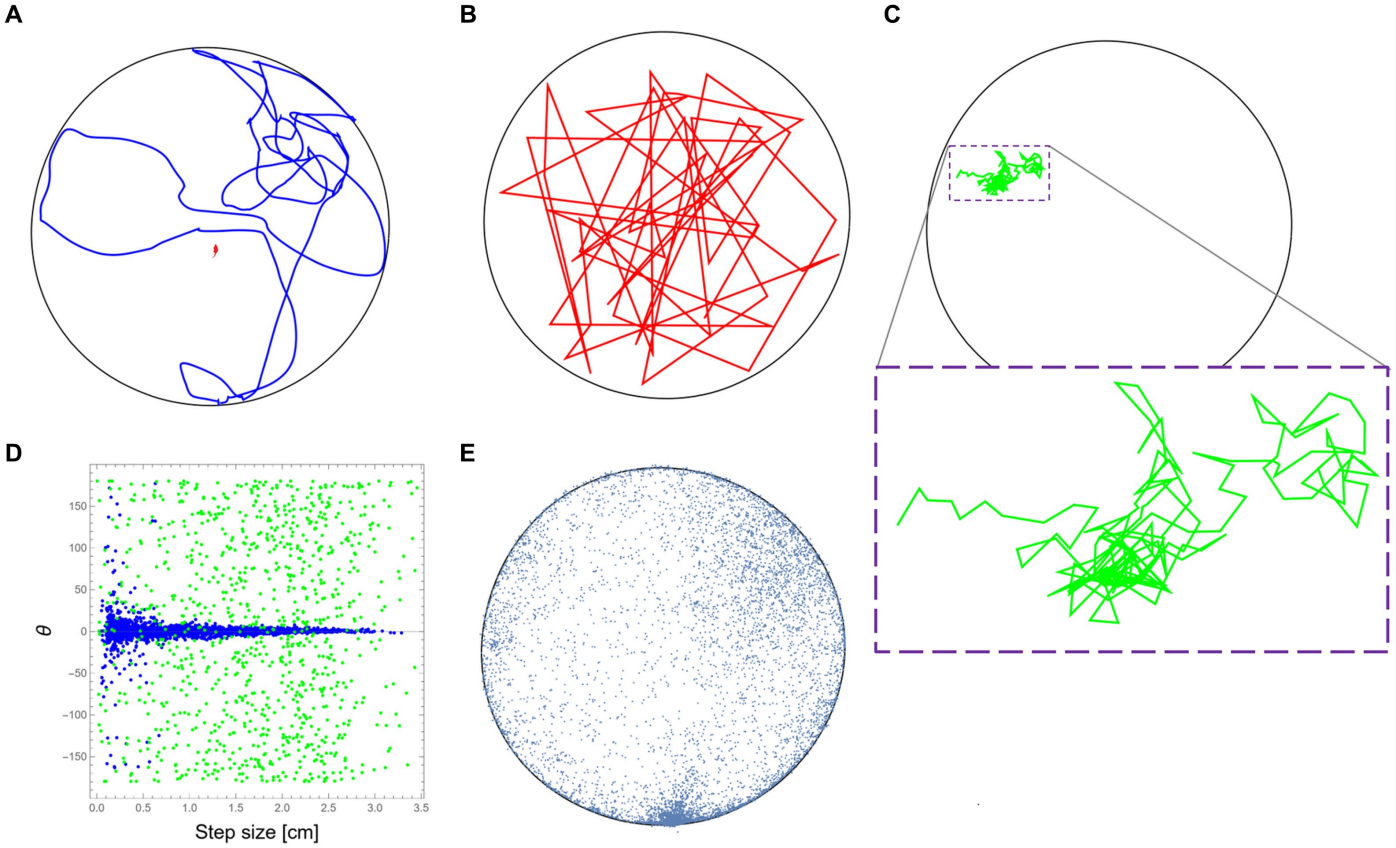
Retaining mousiness by adding constraints. **(A)** A ~ 15 m segment from the original mouse trajectory (in blue) while it was exploring the 2.50 m diameter arena (black circle). **(B)** A path segment was created by connecting random sampling of the original mouse positions without any other constraints (in red). **(C)** A path segment was created after adding the physical constraints of the mouse step size (in green). Due to the small movements, a magnification of the path is provided (dashed purple box). **(D)** The distributions of direction change as a function of step size (speed) for the real mouse behavior (in blue) and the path presented in **(C)** in green. **(E)** A representing sample of the real mouse positions during its exploration of the arena. The frequent visits to the bottom part of the drawing are because that was the location where the home cage of the mouse was attached to the outer wall of the arena.

For this part, we used data from the same study mentioned above (Fonio et al., 2009). The mouse trajectory reflects the natural behavioral signature of the mouse (“mousiness”) while it was moving in the arena (see Figure 1A for a short sample). To demonstrate the idea of retaining the “mousiness” by adding constraints, we first selected a random sample of uniformly distributed positions from the original data set. By connecting these data points in the arbitrary order of the random selection, we formed a new trajectory that can be viewed as the motion of a synthetic mouse that is free of any internal constraints that may exist in the real mouse (Figure 1B). We next started to add specific well-defined constraints to the selection of the data points. For this demonstration, only a few well-known constraints were chosen, but of course, many other constraints, whether known or still unknown, could be added. Here, the first added constraint was the step size and speed due to the anatomy of the mouse, which is on the scale of ~3 cm (Sheppard et al., 2022). This limits the relevant set of data from which we randomly chose the next step. In this way, the constructed trajectory reflects a more constricted synthetic mouse. The next added constraint is the limited change in direction during motion, partially due to inertia. This constraint was based on the measured dependence between step size and change in direction from the original mouse behavior (Figure 1D). This further limits the data set from which we could randomly sample the next step. In this way, a new synthetic mouse can be created, one that is bound both by step size/speed and by the statistics of its ability to deviate its orientation in dependence on its current step size. Finally, the effect of the home cage position was added. This was done by adding to the former constraints the real anisotropic distribution of the visited positions by the mouse in the arena (Figure 1E). Adding this constraint further limits the random sampling of the next steps of the new synthetic mouse. Note that the purpose of these limited examples is only to demonstrate the idea of retaining “mousiness” by adding constraints and does not attempt to replace a worthy full-blown analysis nor to claim that the result presented here is a sufficiently adequate representation of real mouse behavior.

### 2.5 Measuring mirror symmetry

#### 2.5.1 Calculating the symmetry value

**Figure 2:**
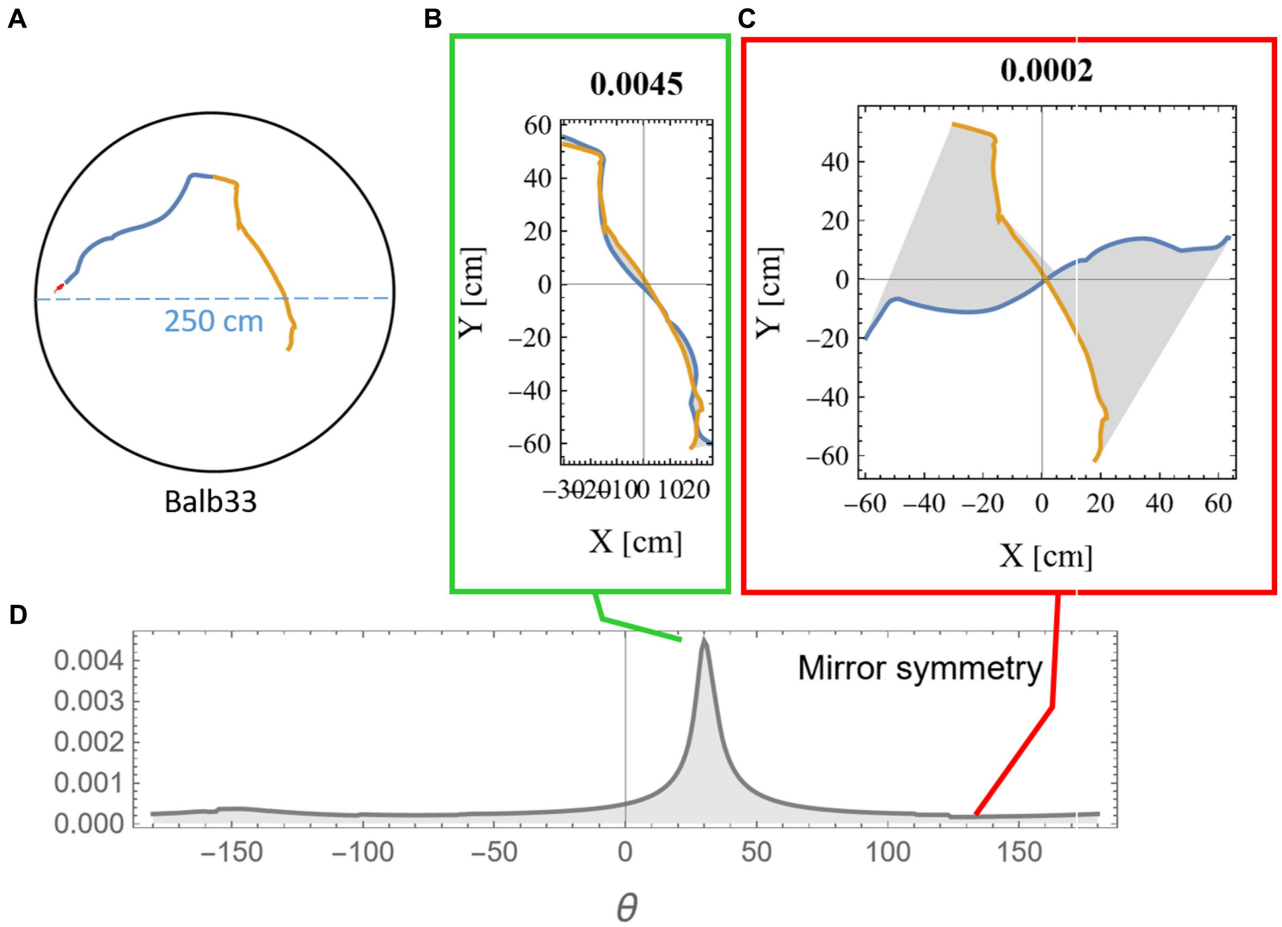
Measuring mirror symmetry in mouse trajectory. **(A)** An example of two consecutive equal-length segments (total length of 280 cm) from a mouse trajectory in a 250-cm-diameter circular arena. The orange segment is the continuation of the blue segment. The tiny red dot at the beginning of the segments represents the real mouse size concerning the arena dimensions. Panels **(B,C)** show the two segments after one was flipped and rotated at 31 and 135 degrees relative to the other. The area between the two curves is colored in gray, and the mirror symmetry score, as calculated in equation (1), is given above. **(D)** The mirror symmetry score for all rotations. The green and red boxes around **(B,C)** point to their corresponding position in **(D)**, where **(B)** corresponds to the peak symmetry value that was selected to represent this specific combination of both points along the trajectory and arc length.

Measuring the mirror symmetry was done by the following computational procedure: (i) two segments of equal length were selected from the mouse trajectory (Figure 2A); (ii) one of the segments was flipped around an arbitrary axis; (iii) the flipped segment was then rotated around its axis in 1-degree steps. For each rotation, we measured the area between the two segments (Figures 2B,C); and (iv) a symmetry score *S* was defined (Equation 1) as a function of the length of the two segments (*l_1_, l_2_*) and the minimal reciprocal area between them *a*(*l_1_, l_2_*) as displayed in gray in Figures 2B,C and explanation in Supplementary Figure S1.


(1) $$S\left(l_{1}l_{2}\right)=\frac{l^{2}}{\min\left(a\left(l_{1},l_{2}\right)\right)}$$

The multiplication by the segment lengths in Equation 1 was necessary to compensate for the expected growth in the gap between the trajectories due to the accumulation of small deviations. The peak value was then chosen as the symmetry score for these two segments (Figure 2D). To demonstrate the statistical significance of our results, we compared symmetry scores between consecutive segments and non-consecutive control segments. All analysis was done using Mathematica (Wolfram Research, Inc., 2023).

#### 2.5.2 Scanning the mouse trajectories

**Figure 3:**
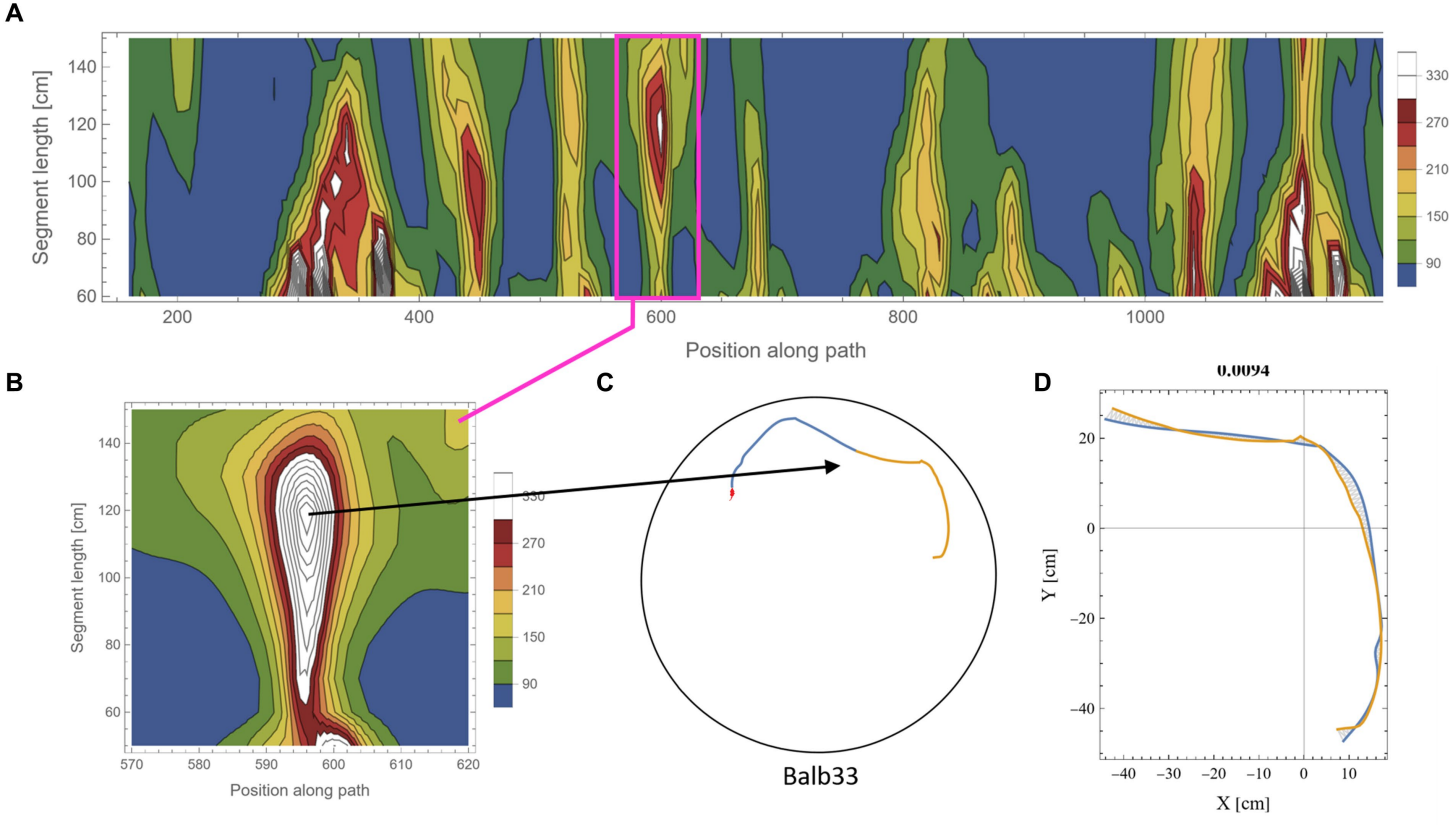
Scanning the mouse trajectory for high mirror symmetry. **(A)** A low-resolution scan of mirror symmetry was calculated by comparing two segments around a central point along the mouse trajectory. The selected central points were 10 cm apart (*x*-axis). For each central point, the symmetry score of two adjacent segments of equal length, ranging between 60 cm and 150 cm (*y*-axis), was measured—denoted by the colored scheme. **(B)** A high-resolution scan of the target range [Magenta rectangle in **(A)**]. Calculations were as in **(A)**, only central points were selected every 1 cm. **(C)** The two successive segments (total length of 240 cm) from the trajectory that corresponds to the peak symmetry value in **(B)**. The tiny red dot at the beginning of the segments represents the real mouse size concerning the arena dimensions. **(D)** The same segments, after flipped one on top of the other and rotated to the best fit (minimal area between lines) demonstrate the high mirror symmetry.

The calculation of symmetry scores, as described above, is computationally costly. To rigorously scan several kilometers of mouse trajectories for high symmetry values and various arc lengths, we first ran a low-resolution scan in which only one data point every 10 cm along the trajectory was used as a point of interest. For each such point, two segments of equal length were taken, and their corresponding symmetry value was calculated as described above. Parts of the trajectory where the mouse moved along the wall of the arena were excluded from the analysis due to the trivial high symmetry in such cases (Supplementary Figure S2). Note that at the beginning of the experiments, the mice showed a gradual build-up in their freedom of movement [reported in detail in Fonio et al. (2009)]. After this relatively short phase, the behavior stabilized for the rest of the test duration (Fonio et al., 2012a,b), and the mice freely explored the full range of the arena, including the central part. Here, we analyzed the mouse behavior only after reaching that level of freedom of movement. The symmetry scores of the scanned trajectories formed arrays that we plotted as contour maps. Such representation somewhat resembles the landscape in a topographic map, where the x-axis represents the position of the reference data point along the trajectory, the *y*-axis represents the range of segment lengths that was used, and the color represents the corresponding symmetry value (Figure 3A, where blue color denotes the lowest symmetry values). The low-resolution scan was good enough to highlight potential areas of relatively high symmetry values that were then scanned at a 10-fold higher resolution, moving 1 cm at a time along the trajectory (Figure 3B). This allowed us to accurately localize [both in the position along the trajectory and the arc length, for which the segments showed the highest symmetry in that region (Figures 3C,D)]. The high-resolution scan was necessary because symmetry values appear to be sensitive to the exact position of the segments along the trajectory, as demonstrated by the typical sharp slope descending from the peak (Figure 3B). Furthermore, note the small size of the mouse relative to the size of the 250 cm diameter arena, demonstrated by the tiny red blob in Figure 3C, representing the actual size of the mouse relative to the arena. Several reasons guided our choice of the selection criteria regarding which parts of the low-resolution scan will be further analyzed in higher resolution: (1) a computational reason: Due to the high computational demands of the scans, there was a need to limit the number of cases that were scanned in high resolution; (2) A conceptual reason: Peak local symmetry comes with a wide range of heights, from shallow “hills” to high “mountains.” If we would like to characterize mountain heights, one may justifiably ask what is the minimum height for an elevated ground area to be considered a mountain. Although there is no clear exact threshold, we could probably agree that given a high enough threshold, all the examples we take can be considered as mountains. Given that this phenomenon has been reported for the first time, we want to restrict ourselves to those examples that show a clear mirror symmetry to a human observer. (3) Finally, a principal justification is derived from the physical scale of the mouse’s body and experimental setup (Figure 2A). This affects two related selection criteria: The first is the segment length—we would probably prefer using arc lengths that are significantly longer than the mouse length but less than the diameter of the arena. The second is the gap between the two segments after folding one on top of the other (Figure 3D). Limiting the analysis to a segment length of about two orders of magnitude larger than the mouse step (about 200 cm) and limiting the inter-segment gap to the mouse length scale greatly reduces the likelihood that high symmetry values will randomly occur. These two factors led to the threshold used in this study (mirror symmetry scores higher than 250).

## 3 Results

### 3.1 Retaining mousiness

To demonstrate the idea of animal uniqueness as the reduction of the potential behavioral space, we start by taking tracked positions of a mouse from a previous study (Fonio et al., 2009) to be our sample database. During this study, Balb/c mice were allowed to freely traverse between the safety of their home cage and a 250-cm diameter empty and novel arena, and their exploratory behavior was recorded (see Methods section). To give some sort of reference to the natural mouse locomotion, an example of a real trajectory of about 15 m long is presented first (Figure 1A). Now, based on the positional data collected from the original mouse, consider an imaginary entity that moves in the same arena and is relieved of all other constraints (Figure 1B). As expected, this imaginary entity randomly jumps around, showing a large variety of velocities and changes in its direction of movement. Unsurprisingly, the result has little to do with the real mouse motion. We then gradually add constraints on the allowed movement that are based on the observed kinematic properties retained from the mouse locomotion. We start by taking mouse anatomy and physiology into consideration, which restricts step size (and reflected in speed) and leads to a highly convoluted path (Figure 1C) relative to the former example. A comparison between the relation of step size and the change in the angle of motion direction between the original trajectory (Figure 1A) and the convoluted trajectory (Figure 1C) demonstrates yet another constraint, probably due to inertia, which further limits the scope of behavioral range by eliminating large changes in the movement direction at higher speeds (Figure 1D).

Examination of a large sample of locations visited by the real mouse (Figure 1E) reveals two further constraints: The first is wall-hugging (Thigmotaxis)—the preference of animals to move close to walls rather than in exposed areas (Barnett, 1963; Simon et al., 1994). The second is the home cage of the mouse that was attached to the outer side of the arena’s wall (see Methods section). As easily noticed, the high density of visited locations in this area (bottom part of the plot in Figure 1E) represents the frequent visits of the mouse to this zone as it moves in and out during its excursions to the arena. Although, in this case, the polarity was introduced as part of the experimental design, the home-base behavior, where the animal has a preferred location relative to which the behavior is organized is well-documented even when there is no apparent objective location (Eilam and Golani, 1989). Despite all the above, even after including some constraints, our imaginary entity is still far from being identified as a genuine mouse. In the discussion, we will briefly mention several more typical constraints on behavior that were discovered over the years during the research in Golani’s laboratory. In the following part, we report an intriguing novel constraint that was discovered during former studies on the exploratory behavior of the same Balb/c mice (Fonio et al., 2009, 2012a,b).

### 3.2 High symmetry in mouse trajectories

**Figure 4:**
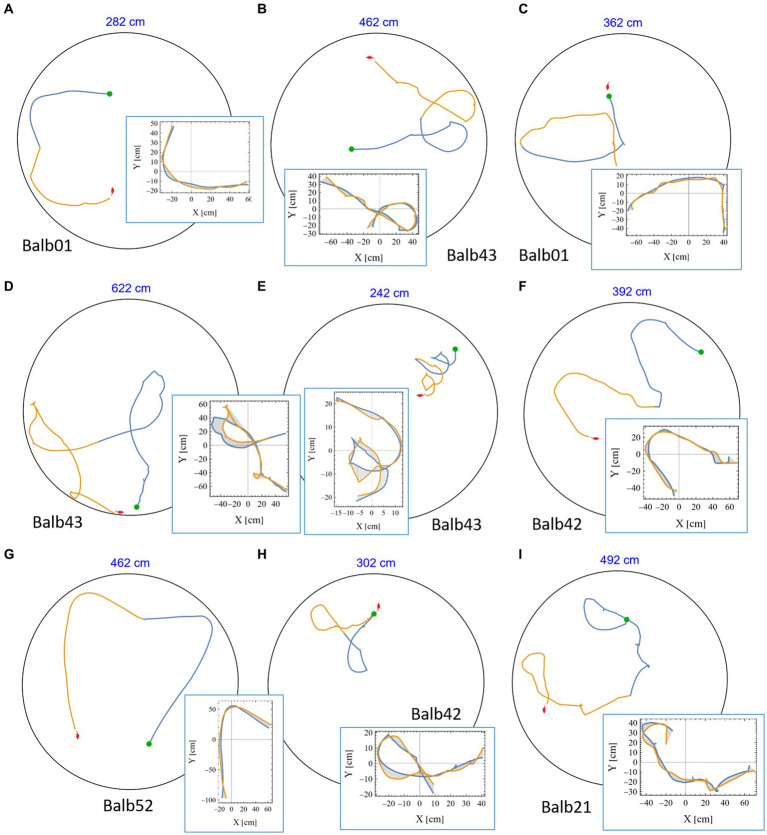
High mirror symmetry examples. **(A-I)** Eight examples of continuous segments from mouse trajectories. Each example includes the relevant path as traveled in the 250 cm diameter arena (black circle), the first half in blue, and the following part in orange, where the total length of the two segments is given above each plot (in blue). Green dots denote the starting position of each pair, and the mouse silhouette (in red), for size reference, was added as well. Each example is accompanied by an inset showing the same two segments, this time where the blue part is folded on top of the orange one. The area between the curves was used as a measure of the level of mirror symmetry (as explained in the Methods section).

A casual review of the mouse trajectories revealed many highly symmetrical segments in which a part of the trajectory was followed by its mirror image. This was found in all tested mice (see representative examples in Figure 4 and Supplementary Figures S3, S4, in which the identity of the mouse is given below each plot). Notably, in many examples, the joint length of the consecutive segments was several meters, and the accuracy of the symmetrical reproduction was so fine that once rotated and drawn one on top of the other, the distance between them was at the scale of few millimeters (insets in Figure 4 and Supplementary Figure S4). In reality, these positions are considerably distant from each other. To appreciate this phenomenon, we plotted the actual size of the mouse silhouette (in red) in Figures 2A, 3C, 4. Highly symmetrical paths can be considered as a limitation on mouse behavior because its future motion seems to be occasionally determined by the path just completed. Knowing that human beings are sensitive to mirror symmetry (Barlow and Reeves, 1978; Treder, 2010), the question of whether these occurrences reflect a real phenomenon at all was raised. To answer this concern, we set our goal to rigorously search for highly symmetrical path segments and compare the symmetry value of adjacent trajectories to that of randomly selected parts from the same mouse as a control.

#### 3.2.1 Scanning for high symmetry in mouse trajectory

The Methods section includes a detailed explanation of the procedure of searching for high symmetry along the mouse trajectory. In short, more than 2,500 m of mouse locomotion were scanned at low resolution: We conducted a 2D scan (at a resolution of 10 cm) over both the starting point of the segments and their arc length (Figure 2A). To measure mirror symmetry, one of the segments was first flipped and then rotated against the other (Figures 2B,C) until the angle that minimizes the area between the trajectories was identified (Figure 2D). The symmetry score was chosen to be high for small areas.

**Figure 5:**
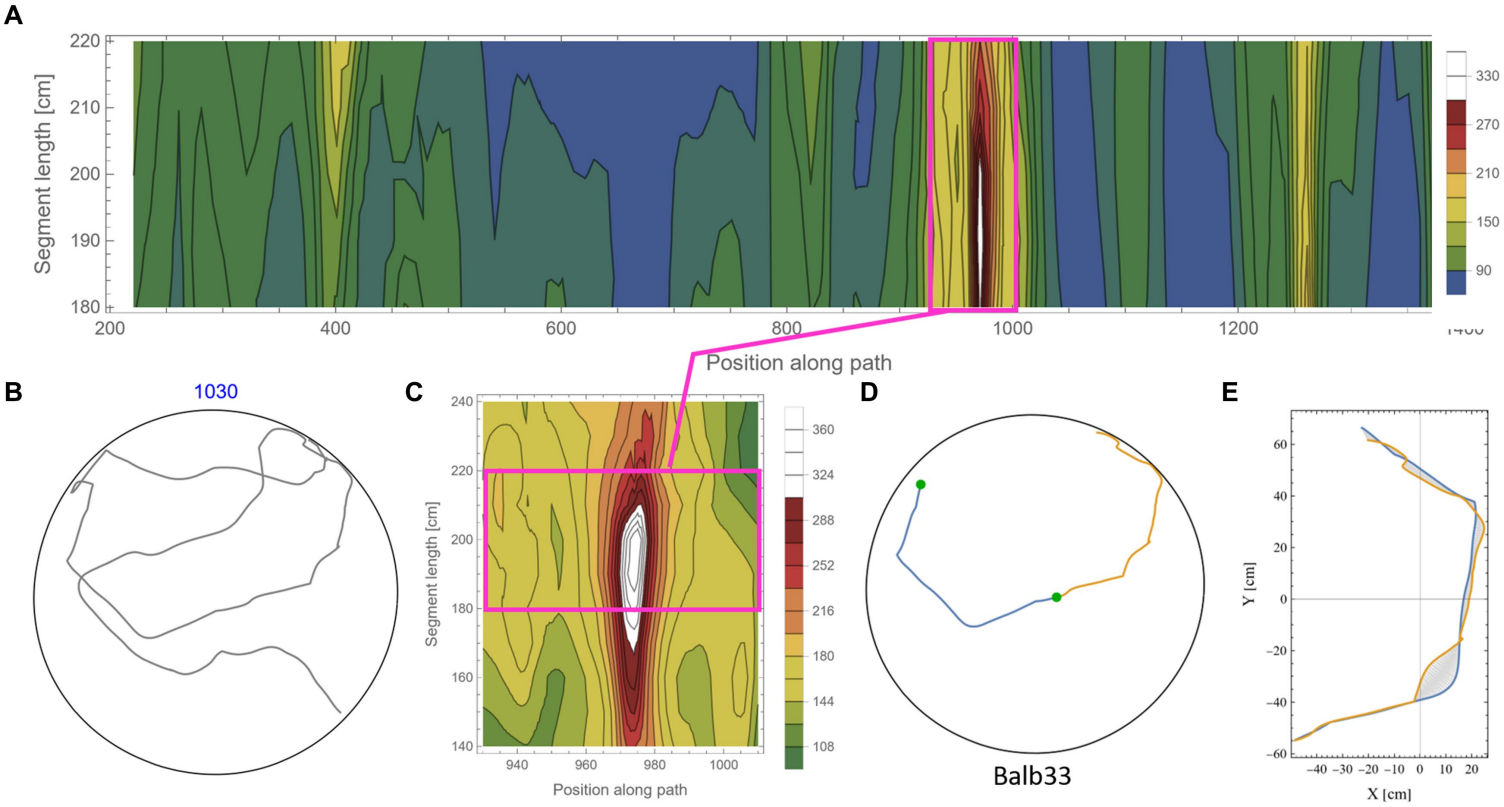
Scanning for high mirror symmetry in longer segments. **(A)** A low-resolution symmetry scan of **(B)** a ~ 10.3 m trajectory. **(C)** A high-resolution scan of an isolated island of high symmetry. **(D)** The two successive segments (total length of 380 cm) from the original trajectory **(B)** correspond to the peak symmetry value in **(C)**. **(E)** The same segments are centered at the axes’ origins and rotated to the best fit (minimal area between lines), demonstrating the high mirror symmetry.

Figure 5 shows an example of the rigorous scan for high symmetry values along the mouse trajectory for segments in the range of 180–220 cm (each). From the low-resolution scan (Figure 5A) of a trajectory (Figure 5B), an island of high symmetry values was detected and further scanned at a higher resolution (Figure 5C). Note that the island of high symmetry sometimes expanded beyond the specified range of the low-resolution scan. In such cases, the range was extended in the high-resolution scan to ensure that the peak symmetry value is found (Figure 5 and also in Supplementary Figure S5). Once the local maximum value was found, a visual inspection of the specific segments (Figure 5D) and the minimal area between the two segments (Figure 5E) was made. Islands of relatively high symmetries varied considerably in their peak value. To avoid the question of what “nice” symmetry is, an arbitrary threshold of 250 was set (see scale in Figures 5A,C). In this way, only prominent examples of symmetry were scanned in high resolution and were included in the analysis. This also kept the large computational cost of the additional high-resolution scan in a reasonable range. The arbitrarily high threshold also relieved us from another concern regarding how to address high symmetry scores in overlapping short segments along the trajectory (see an example in Supplementary Figure S6). In total, the percentage of arc length showing high symmetry values out of the total arc length of the analyzed trajectory is ~11.3%. Note that this calculation included only half of the arc length, which shows high symmetry. In other words, taking into account both parts that belong to high symmetrical segments, the percentage is 22.6% out of the total analyzed trajectory. Actually, the high symmetry is expected to be even more prevalent in shorter segments, as can be seen in the examples given in Figure 3 and Supplementary Figure S6.

#### 3.2.2 Scanning for high symmetry in temporally separated segments

**Figure 6:**
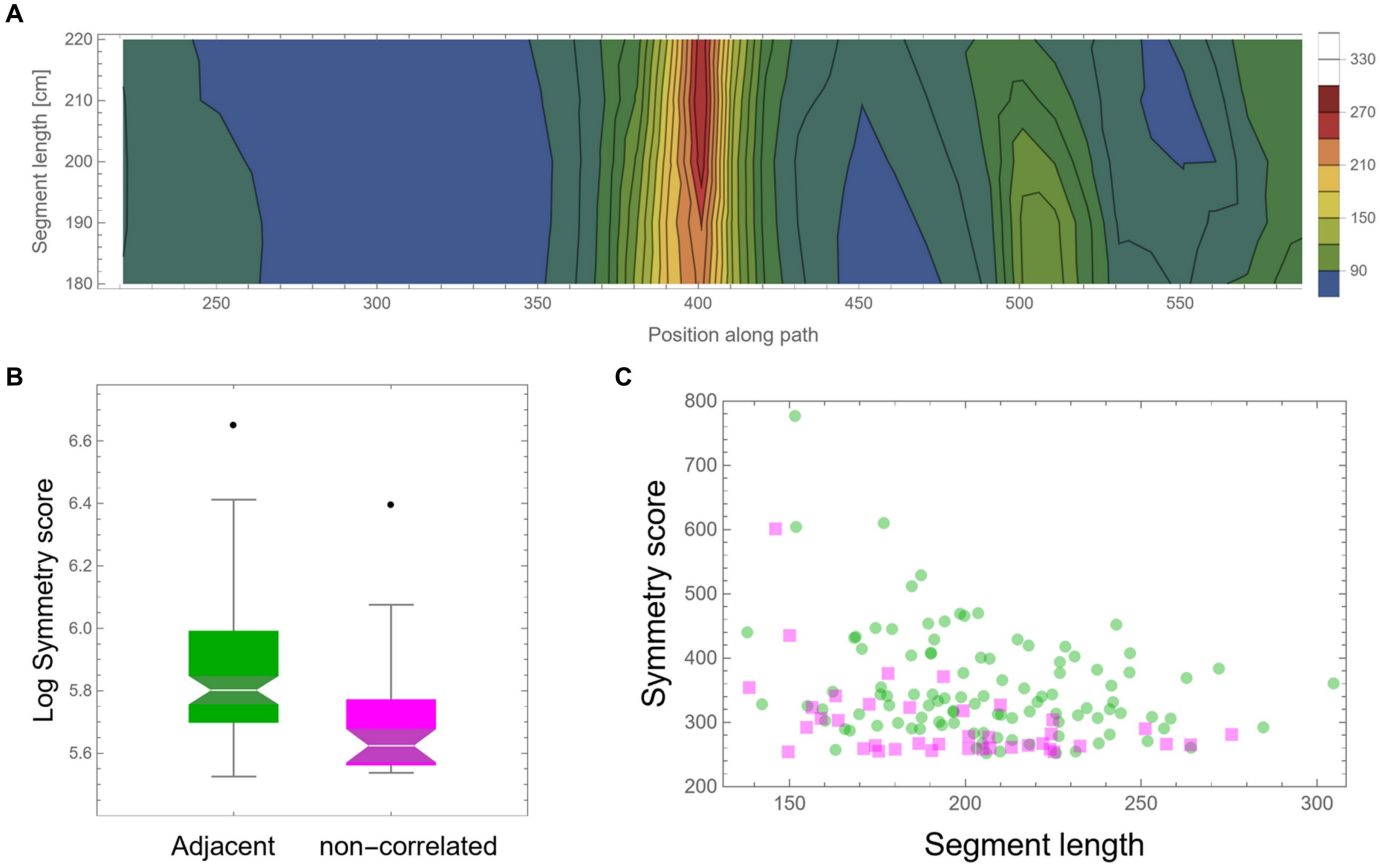
Control—fixed random time lag. **(A)** A low-resolution symmetry scan of segments that are separated by a fixed random time lag (hundreds of meters apart). **(B)** A box-plot comparison of peak symmetry values for adjacent segments (in green) and non-correlated segments (in magenta) shows a significant difference (*p*-value <0.00002). Scores were transformed using log scaling to shift the distribution toward normality. **(C)** Peak symmetry scores of the same data are presented in **(B)** as a function of segment length. One hundred five instances were found for adjacent segments and only 39 for the non-adjacent scan.

As mentioned above, the high symmetry found might be an artifact that one can expect to appear by mere chance in such large datasets. Another possible simple explanation could be that high symmetry is a general property of the locomotion of these mice, perhaps due to low motor “vocabulary.” In such cases, the mice have only a few motor plans that they can execute; hence, frequent high symmetrical patterns will unavoidably appear. Note that in both cases, we should not expect that high symmetry values will exclusively appear in consecutive segments; rather, we expect to find similar high values, and at the same rate, also between segments that are temporally separated. Moreover, dull motor repertoire would also suggest that different highly symmetrical curves would frequently show high similarity in their shape. To examine these possible explanations, a similar scan was performed; only this time, the two trajectory segments were separated by a fixed random time lag. Other than this, all further analysis was identically applied as described above. A similar total path length (more than an additional 2,000 m) of mouse trajectory was scanned in this way as control. Figure 6A shows an example of a low-resolution scan of such dissociated pairs of segments, showing an example of an island of relatively high symmetry value with a peak at ~210 cm segment length each. A comparison of all peak symmetry values that were found in both conditions, either for successive segments or segments with random temporal gaps, is presented in Figure 6B. Successive segments show significantly higher mirror symmetry values (*t*-test: *p*-value <0.00002). Although the total trajectory length that was scanned for both conditions was similar, there were 2.5 times more high symmetry events for successive segments (105 cases) than for non-adjacent segments, for which only 39 cases were found (Figure 6C).

Suspecting that the separation of segments, applied in the above control method, might somehow hamper the appearance of high symmetry (e.g., due to the discontinuity of the segments), we checked this concern by simulating trajectories based on the spectral properties of the original mouse behavior (see Supplementary Results—spectral-based simulations in the supplementary information). The results presented in Supplementary Figure S11 show that there is no significant difference in the frequency of high symmetry values in simulated trajectories (see ANOVA test in Supplementary Table S1). This shows that the separation method used for the control cannot explain the significant difference presented in Figure 6B. Note that there is also no significant difference between the spectral-based simulations and the lagged segments in the original control. This further supports the claim that whereas part of the high symmetry could, in principle, be attributed to the random spectral properties of the behavior of these mice, it cannot explain the significantly higher level of symmetry that exists in adjacent segments (Figure 6B and Supplementary Figure S11). In addition, as can be viewed in the various examples given in Figures 3–5, as well as in Supplementary Figures S3–S5, highly symmetrical segments show a large variety of shapes. Taking all together, these results confirm that the high symmetry in successive segments cannot be explained by mere chance, low motor “vocabulary,” or any other suggestion that does not involve temporal proximity in its mechanism.

## 4 Discussion

In this study, we addressed some of the ideas that are presented in the recent publication titled: “On the growth and form of animal behavior” (Golani, 2022). Specifically, we wish to expand on the realization that various constraints have an impact on the expressed behavior. We therefore suggest the use of constraints and limitations as a methodical complementary approach to descriptive characterization of the organism’s freedom of operational space. Comparable to the artist painter, carefully adding strokes of paint on the white canvas, the sculpture carves its way to a target image by incrementally removing parts from a slab of marble. Both the empty canvas and the untouched stone hold an endless potential of outcomes. The difference is that the painter positively adds the features of the image, whereas the sculpture gradually adds constraints by removing those parts that do not belong to the result. Although not common, definitions of absence do exist in various scientific fields (Chapple et al., 2018; Gourlay, 2022).

We argue that exposing the constraints applied to the animal could be an interesting methodology to sculpt the behavioral space. By finding all relevant limitations to a certain creature, say a mouse, we regain a deep understanding of its unique mousiness. In the first part of the results, we presented a somewhat simplified demonstration of this idea and showed that by adding some elementary physical constraints, we can vastly reduce the wide range of potential behavior (Figures 1C,D). We further mention non-physical constraints (Figure 1E) related to known limitations of mouse exploration due to the avoidance of exposed space and the anisotropic effects of home-based behavior (Eilam and Golani, 1989). Note that in contrast to the definite physical constraints, the latter examples demonstrate that the reduction of behavioral space could also be manifested in the probability of the occurrence of behaviors by reducing the likelihood of their appearance. We ended our demonstration only after a few steps; however, many other constraints were clearly not mentioned. For example, higher kinematic derivative dependencies, such as in motion curvature, acceleration, and jerk (Hogan and Flash, 1987; Viviani and Flash, 1995; Zago et al., 2018), can also be considered. In fact, during decades of behavioral research in Golani’s lab, many more constraints were discovered as part of the quest to find an objective structure. Structure inherently implies constraints. A partial list would include the existence of speed modes that define stopping behavior and motion episodes between places in specific speed modes (Drai et al., 2000, 2001) an upper bound on the number of stops during excursions (Golani et al., 1993) as well as many other discoveries in vertebrates, mostly in rodents (Golani et al., 1993; Tchernichovski and Golani, 1995, Kafkafi and Golani, 1998; Kafkafi et al., 2003; Lipkind et al., 2004; Fonio et al., 2006, 2009, 2012a,b; Gruntman et al., 2007; Horev et al., 2007; Dvorkin et al., 2008; Benjamini et al., 2011; Wexler et al., 2018) as well as in invertebrates such as flies (Gakamsky et al., 2013; Cohen et al., 2015). Constraints play an important role also in the description of animal-to-animal interactions, where, for example, inferiority is expressed in the reduction of freedom of movement (Golani and Moran, 1983; Yaniv and Golani, 1987). Here, we only intended to demonstrate the feasibility of the construction of synthetic trajectories by adding constraints to a disconnected sample of positional data from a real animal trajectory. Implementing this approach to find novel constraints requires devising a metric of similarity so one can properly quantify the multidimensional behavioral space as it shrinks due to the addition of constraints. Such a metric could be derived using AI technology, for example, from how well it distinguishes simulated trajectories from real ones (Tao et al., 2021).

Following the general demonstration of adding constraints as a way to regain, in this case, the mousiness nature, we showed a novel constraint manifested as high mirror symmetry in the mouse movement trajectories. The content of symmetrical patterns can be compressed due to the redundancy of the relevant information (Bairagi, 2015). Somewhat similar to the “last in first out” rule discussed by Golani concerning the restriction of the freedom of movement due to, e.g., stress (Golani, 2022), mirroring movement means that the recently executed motor plan is reproduced in a reversed order, thus adding limitations on the expressed behavior and reduces computational demand. Considering that the step size of mice is on the scale of ~3 cm (Sheppard et al., 2022), the observed high symmetrical trajectories, often spanning for several meters, are surprising, to say the least. A possible mechanism for this phenomenon may be the principle of reversed replay of neuronal activity, where the same neuron activity monitored from the brain during motion is triggered again in reverse order during inactive periods that follow. This pattern was found in various brain areas and neuron types (Eichenlaub et al., 2020), especially in place cells in rats, both in linear tracks (Foster and Wilson, 2006; Diba and Buzsáki, 2007) and during exploration in the open field (Csicsvari et al., 2007), and also in the human brains (Eichenlaub et al., 2020). These observations were suggested to be related to memory consolidation and learning of recent events (Foster and Wilson, 2006; Carr et al., 2011). Although this is a highly speculative suggestion, if the principle of reversal neuronal activity is applied such that the coded motion of the animal during a movement segment is reversibly reproduced, this could theoretically create an adjacent movement segment, which is the mirror image of the preceding one. This hypothesis may indicate a prediction that in those cases where the replay activity is recorded during an inactive phase, there should be a parallel inhibition in the motor cortex that, in our case of motion segments showing high mirror symmetry, is not activated.

Although constraints lead to the reduction of behavioral space, they do not reduce the complexity of the observed behavioral repertoire. On the contrary, as in life itself, where the complexity of behavior thrives somewhere in between random noise and low information patterns (Mayer, 2020), so does the animal’s behavioral space. Too limited repertoire may indicate a pathological state. On the other hand, non-controllable motion, although it includes, in principle, all possible behaviors, effectively produces only random noisy patterns in any reasonable period. It is the reduction in degrees of freedom that enables the emergence of controllable and purposeful patterns (Marken, 1991; Hochner et al., 2023). As mentioned above, some of these constraints have nothing to do with the straightforward physicality of the animal. Rather, these constraints are related to cognitive (Shettleworth, 2001; Stevens and Stevens, 2012) and emotional (Archer, 1973) schemes. By accounting for all these limitations, one should be able to incrementally retain the objective fine structure of behavioral space that is unique to the animal. The generality of this approach makes it potentially useful in searching for commonalities in various organisms across taxa, such that their behavior is expressed as motion trajectories. In addition, by inducing manipulations on internal states, exploring various motivational aspects (such as hunger), inducing stress, or testing the animals in more cognitively demanding tasks, one can use this method to decipher how the change in the internal constraints affects the properties of the expressed behavior, in this case, the high mirror symmetry.

The high symmetry phenomenon reported here was discovered while testing mice in a large and empty arena. One could wonder whether such high mirror symmetry will also occur in more natural conditions. The fact that this behavior did not emerge due to external constraints indicates that it reflects the intrinsic preferences of the behaving animal. Although we believe that such internal schemes may also appear in more enriched environments, we suspect that this particular phenomenon might be obscured in such conditions. Studying stopping behavior, for example, reveals that in an enriched cage full of various objects, mice will tend to move between objects and stop in their vicinity (Könings et al., 2019). Only when observed in a largely empty arena, a condition that is not so unrealistic for mice, one would discover that structured stopping behavior is a fundamental intrinsic property of these animals, which is expressed even without external stimuli (Eilam and Golani, 1989; Golani et al., 1993). The enriched environment, in this case, obscured the internal variables that the animal is controlling.

While ethological research aims to study animal behavior in a natural environment, it is also highly informative to expose natural intrinsic constraints, which could be more intriguing than the somewhat trivial finding that external environmental properties can shape animal behavior. Exposing intrinsic constraints can potentially lead to further investigation of the related underlying mechanisms, such as neuronal constructs. In the same way, here, high symmetry movement along the curved wall reflects a trivial behavior due to the external constraint of the environment (Supplementary Figure S2), whereas the high symmetry on which we report is surprising, exposing a non-trivial intrinsic property of these mice.

Once the symmetry measure is set, one can apply it to trajectories of other strains and in various conditions. A preliminary analysis of two other mouse strains that were tested in the same experimental setup, c57BL6 [described in Fonio et al. (2009)] and wild mice [described in Fonio et al. (2012a,b)], revealed a similar percentage of high mirror symmetry along the mouse trajectory (see in the Supplementary Results). We noticed, however, that whereas the high symmetrical motion segments produced by the Balb/c mice are frequently complex and convoluted (as can be seen in the many examples given in Figure 4 and Supplementary Figures S3–S5), such segments of the other strains seem to be simpler and more predictable (Supplementary Figures S6, S7). Similar results are apparent in the motion segments of the control test (Supplementary Figure S9), where non-temporally correlated segments were examined. Although this should be further studied and properly quantified (maybe by adding a measure of complexity to the trajectory), the qualitative difference is clear, suggesting that the symmetry found in adjacent motion segments by the Balb/c mice is more profound and less likely to occur by chance. Taking all together, the results suggest that this phenomenon is not strain-specific but rather a characteristic of mice in general. Further examination of other taxa and other conditions is needed to reveal to what extent this behavior is preserved.

Finally, the novel phenomenon of high mirror symmetry in the mouse trajectories presented here demonstrates yet another important role of the ethological study, which goes beyond the study of behavior itself. We argue that such ethological studies may be indispensable to other research fields because behavioral constraints have a strong genetic and neurological basis. This gives the scientific community a promising source of well-defined behavioral phenomena to which they can apply their advanced tools and knowledge. Therefore, the identification of behavioral constraints as a methodology to decipher the structure of behavior should become part of our toolbox for behavioral studies.

## Data availability statement

The raw data supporting the conclusions of this article will be made available by the authors, without undue reservation.

## Ethics statement

The animal study was approved by Tel Aviv University Institutional Animal Care and Use Committee (IACUC) - Assurance Number A5010–01. The study was conducted in accordance with the local legislation and institutional requirements.

## Author contributions

EF: Conceptualization, Formal analysis, Investigation, Methodology, Project administration, Visualization, Writing – original draft, Writing – review & editing. OF: Conceptualization, Methodology, Writing – original draft, Writing – review & editing.
