# Supplementary material for: High mirror symmetry in mouse exploratory behavior
Source: Front Behav Neurosci. 2024 Apr 29;18:1381852. doi: 10.3389/fnbeh.2024.1381852 (PMC11089150; doi:10.3389/fnbeh.2024.1381852)
Supplement: Supplementary file 1 [file Data_Sheet_1.docx]

Supplementary Material

# Supplementary Figures and data

## Supplementary Figures


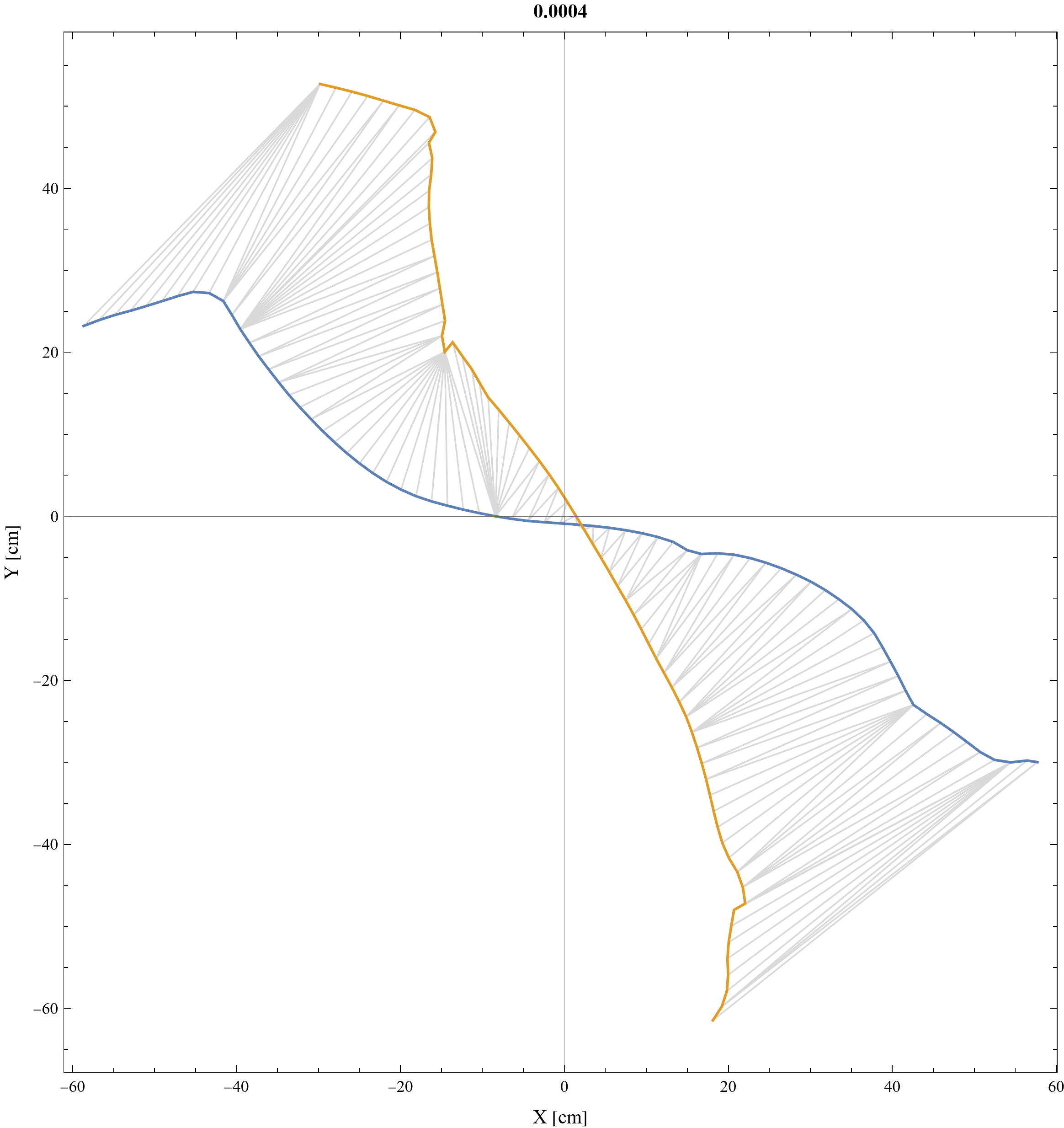


**Supplementary Figure S1.** The area between segments was calculated as the sum of all the non-overlapping triangles formed by two successive data points on one segment and one data point on the other segment. The reciprocal value of this total area is given above the image.


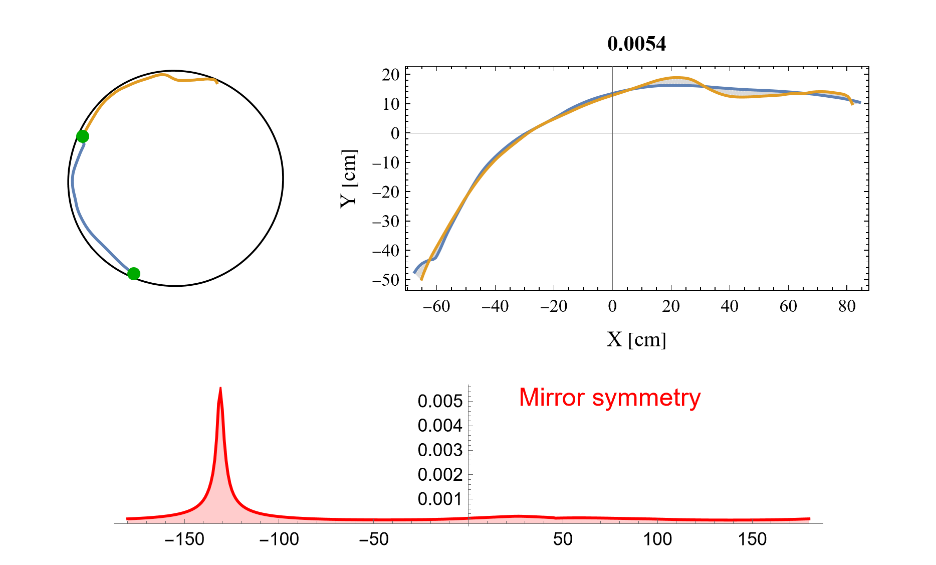


**Supplementary Figure S2.** Trivial high symmetry during wall movement: **(A)** Mouse trajectory segment of movement along the wall of the arena. **(B)** The high fit between the two consecutive segments when plotted flipped one on top of the other (see methods). **(C)** The calculated reciprocal area value between the 2 segments for various rotation angles. The peak corresponding to the rotation presented in (B) suggests a high mirror symmetry score for these segments.


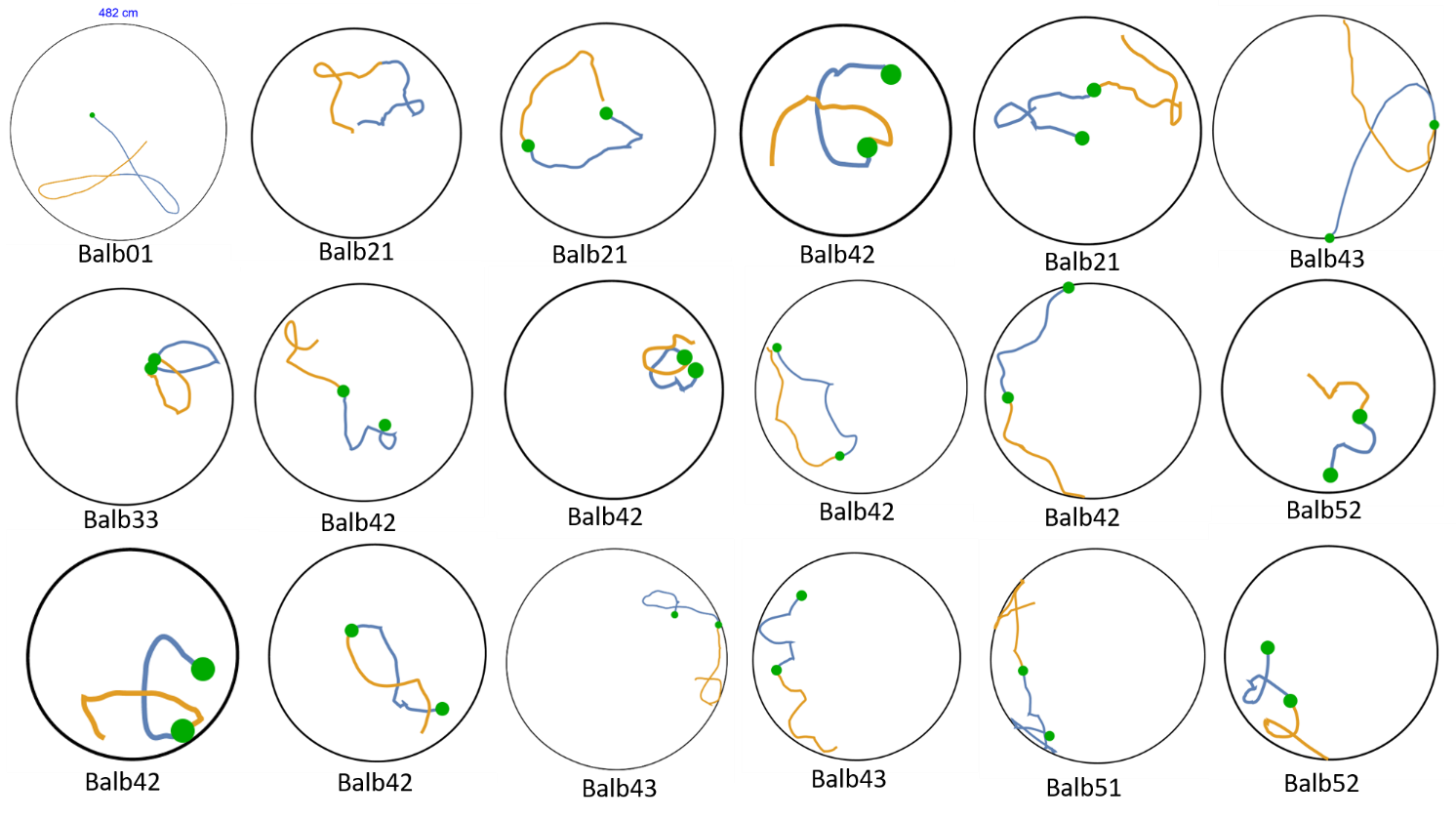


**Supplementary Figure S3.** 18 more examples of continuous segments from mouse trajectories, showing high mirror symmetry. Each example shows the relevant path as traveled in the 250 cm diameter arena (black circle), the first half in blue and the following part in orange. Green dots denote the starting position of each pair. For scaling reference, the length of the trajectory in the first example (top left) is given in blue.


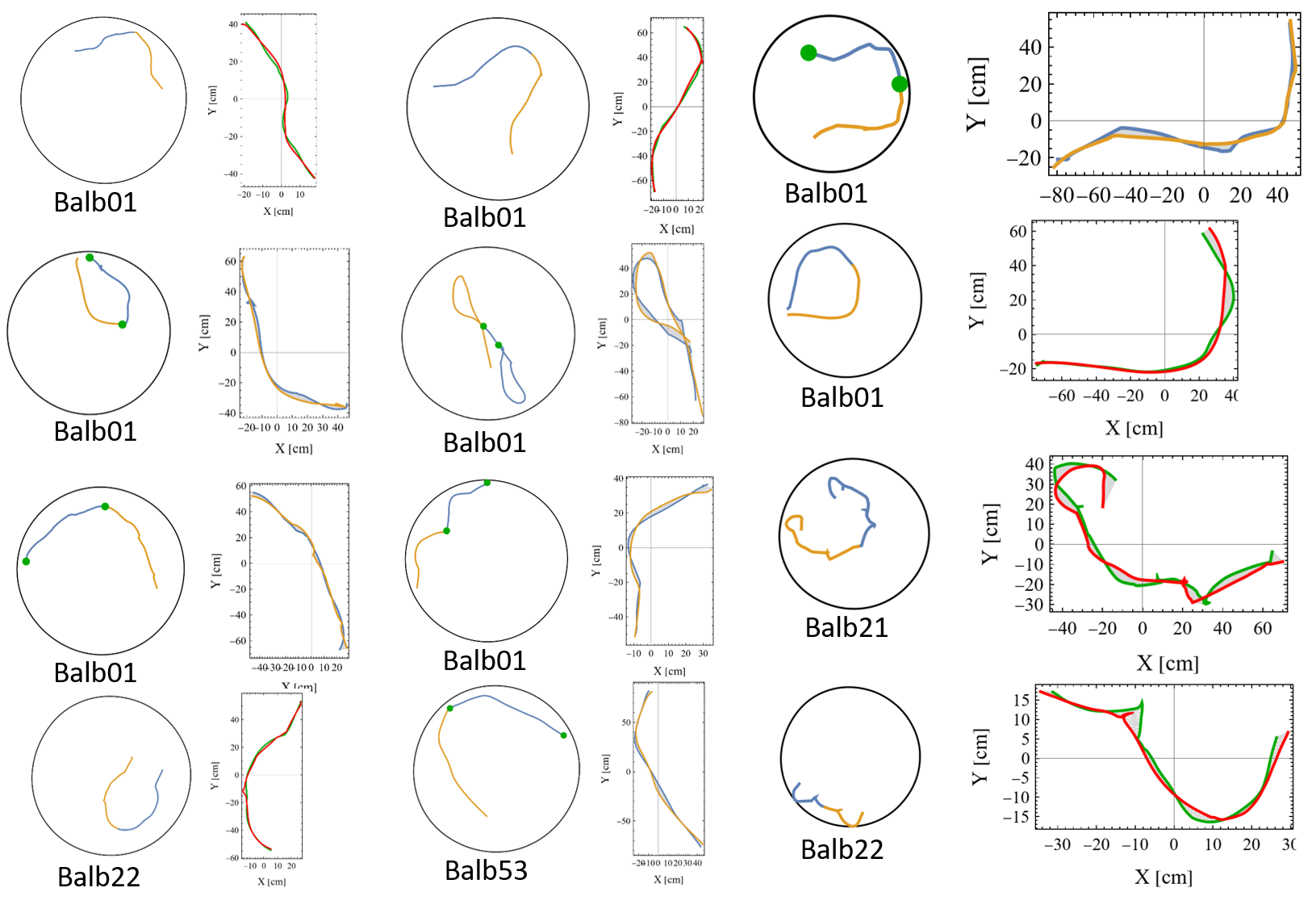


**Supplementary Figure S4.** 12 more examples of high mirror symmetry with remarkable fit: Each example includes the relevant path as traveled in the 250 cm diameter arena (black circle), first half in blue and the following part in orange. The total length of the 2 segments is given above each plot (in blue). Green dots denote the starting position of each pair. Each example is accompanied by an inset showing the same 2 segments, this time where the blue part is folded on top of the orange one. The area between the curves was used as a measure of the level of mirror symmetry (as explained in the methods section).


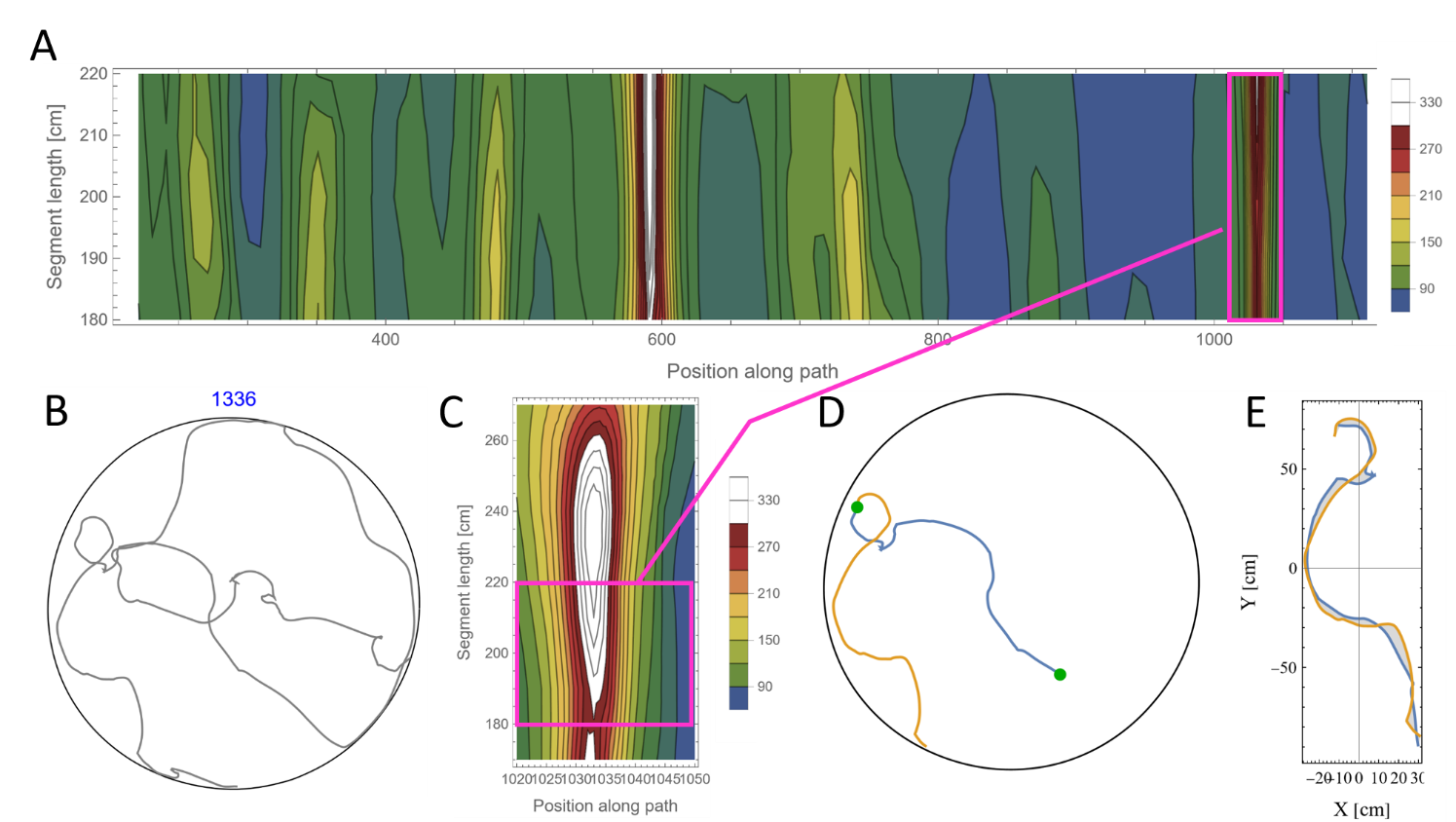


**Supplementary Figure S5. Widening the range of high-resolution scan: (A)** A low-resolution symmetry scan of **(B)** a ~10.3 m trajectory. **(C)** A high-resolution scan of an isolated island of high symmetry (marked in pink). The high-resolution scan included a wider range to ensure finding the peak symmetry value. **(D)** The two successive segments (total length of 460 cm) from the original trajectory (B) that correspond to the peak symmetry value in (C). **(E)** The same segments after centered at the axes' origins and rotated to the best fit (minimal area between lines) demonstrating the high mirror symmetry.

After scanning segment lengths ranging between 60-150cm (for each part of the examined pair), we find that high mirror symmetry is so abundant in segments within this range that the landscape that appeared in the scan plots was often a compound of nearby connected peaks as well as overlapping segments (Figure 2A and Supplementary Figure 6A). This introduced potential redundancy and ambiguity in the selection of the high symmetrical cases. To objectively select well-defined high symmetry scores, the mouse trajectory was rescanned by using an even higher range of segment lengths.


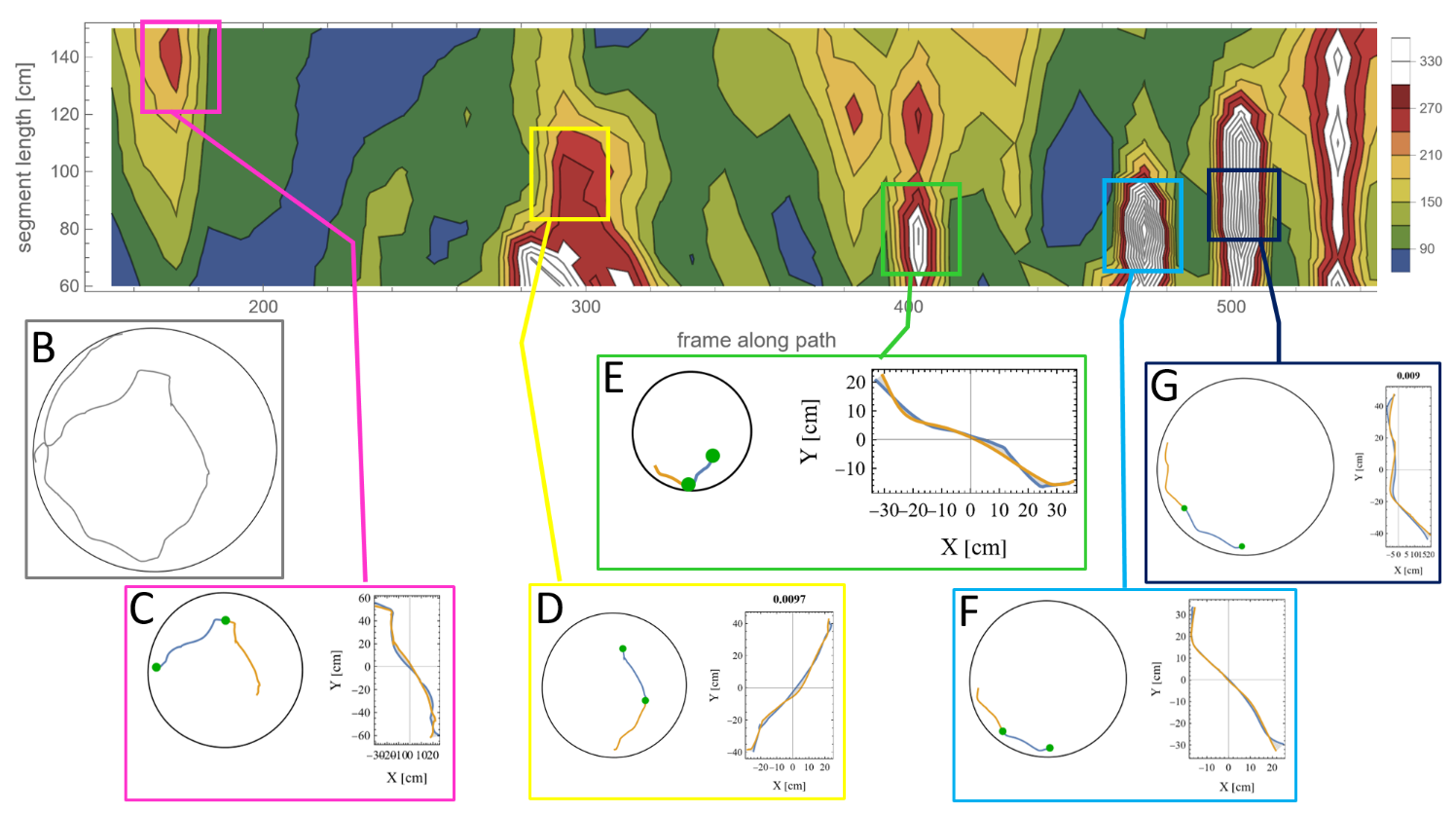


**Supplementary Figure S6.** Overlapping high mirror symmetry: (**A**) A low-resolution symmetry scan of a ~7 m trajectory that is presented in (**B**). The color bar signifies the symmetry score. (**C-G**) Overlapping examples of high mirror symmetry segments along the trajectory presented in (B) that correspond to local peaks in the original scan (A).

## Supplementary Results – High symmetry in other mouse strains

Once the symmetry score is defined, it can be easily applied to trajectories of other strains and in other conditions. A preliminary analysis of two other mouse strains that were tested in the same experimental set-up: c57BL6 (described in Fonio et al., 2009) and wild mice (described in Fonio et al., 2012), revealed a similar percentage of high mirror symmetry along the mouse trajectory. The c57BL6 strain showed 11.1% (out of a total of 612m analyzed) and the wild mice showed 8.1% (out of a total of 585m analyzed). Supplementary figures 7 and 8 present the high mirror symmetry examples found during the preliminary scan.


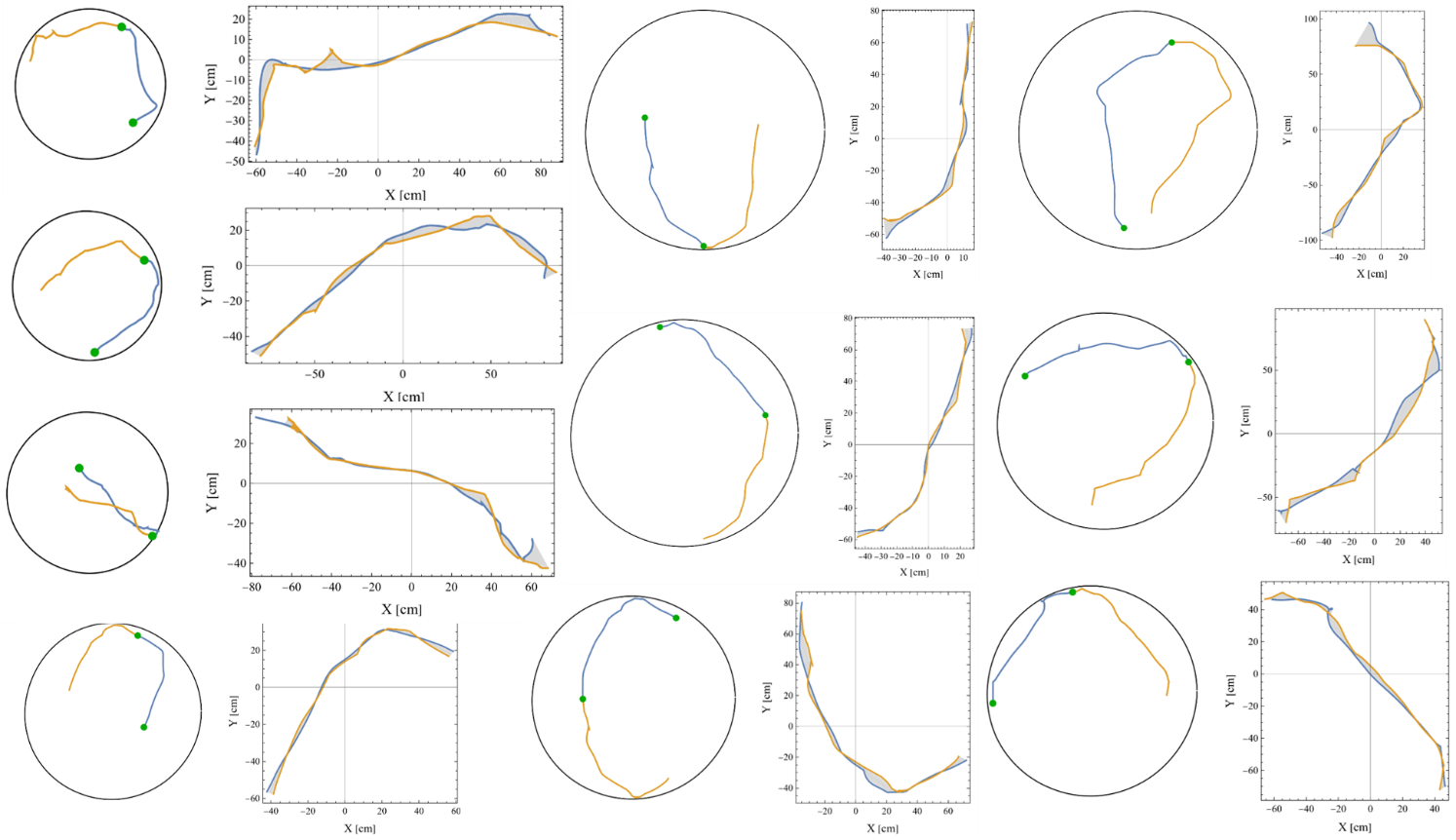


**Supplementary Figure S7.** 10 examples of high mirror symmetry from the exploratory behavior of C57BL6 mice in the same experimental conditions: Each example includes the relevant path as traveled in the 250 cm diameter arena (black circle), the first half in blue, and the following part in orange. The total length of the 2 segments is around 4m for all examples. Green dots denote the starting position of each pair. Each example is accompanied by an inset showing the same 2 segments, this time where the blue part is folded on top of the orange one. The area between the curves was used as a measure of the level of mirror symmetry (as explained in the methods section).


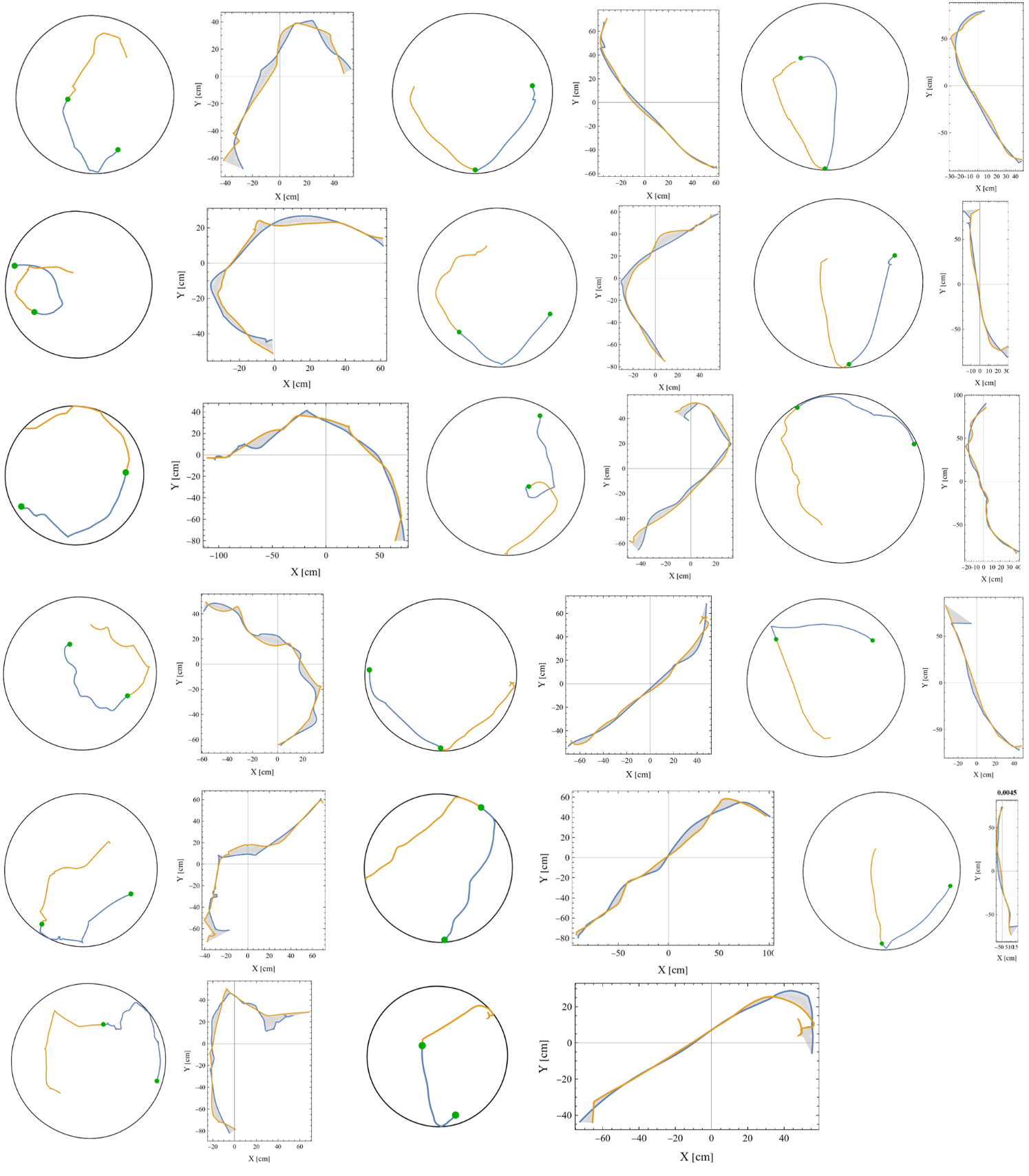


**Supplementary Figure S8.** 17 examples of high mirror symmetry from the exploratory behavior of wild mice in the same experimental conditions: the first half in blue and the following part in orange. The total length of the 2 segments is around 4m for all examples. Green dots denote the starting position of each pair. Each example is accompanied by an inset showing the same 2 segments, this time where the blue part is folded on top of the orange one. The area between the curves was used as a measure of the level of mirror symmetry (as explained in the methods section).

**
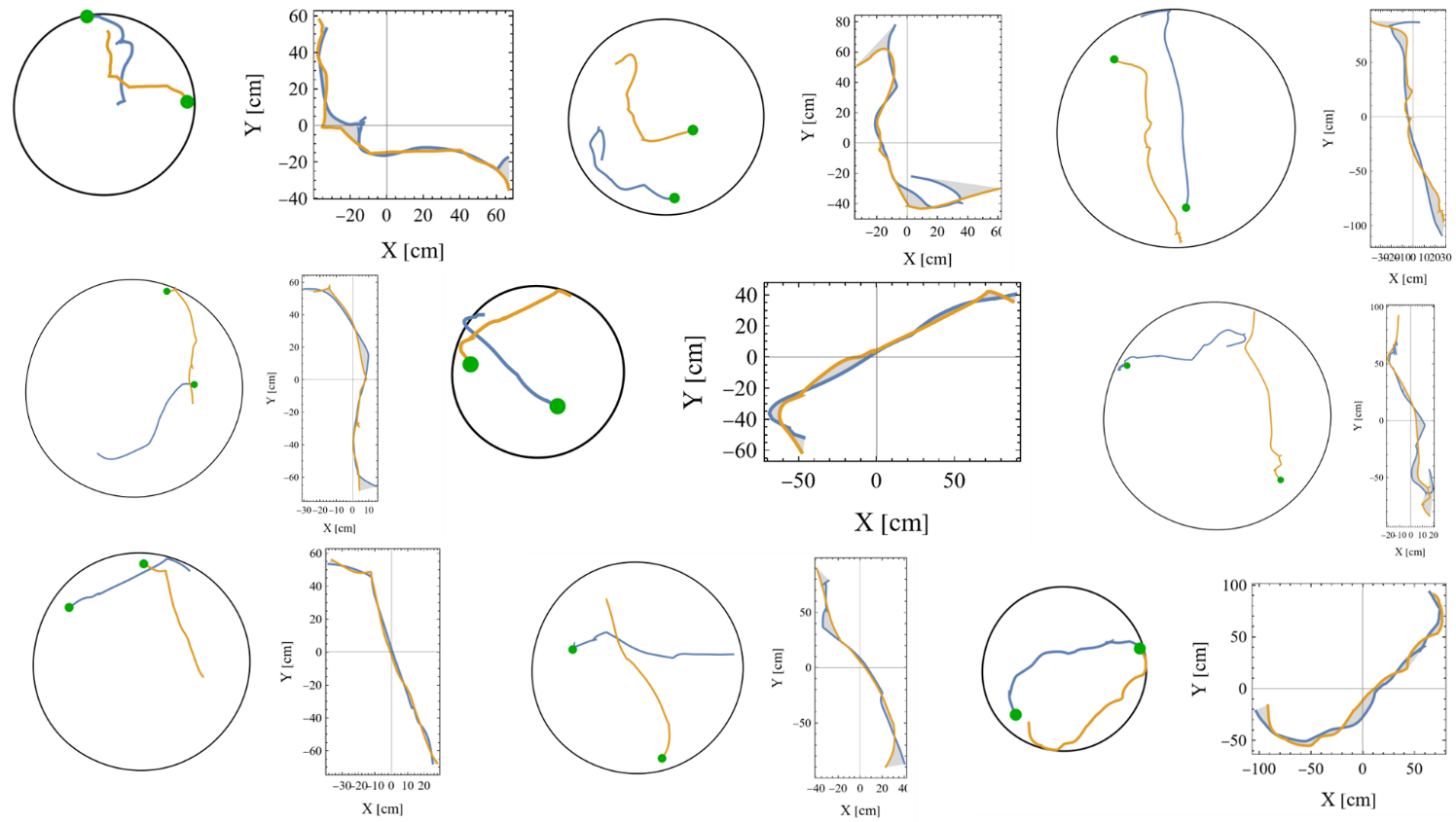
**

**Supplementary Figure S9.** 9 examples of high mirror symmetry from the exploratory behavior of Balb/c mice in the same experimental conditions: Each example includes two segments separated by a randomly selected time gap as traveled in the 250 cm diameter arena (black circle), one segment in blue and another in orange. The total length of the 2 segments is around 4m for all examples. Green dots denote the starting position of each pair. Each example is accompanied by an inset showing the same 2 segments, this time where the blue part is folded on top of the orange one. The area between the curves was used as a measure of the level of mirror symmetry (as explained in the methods section).

## Supplementary Results – spectral-based simulations

To simulate the general kinematic properties of the mouse behavior we performed spectral analysis of real mouse trajectory. We then used a reversed spectral analysis to create trajectories that simulate the original behavior (see below the function that we used in Mathematica programming environment).


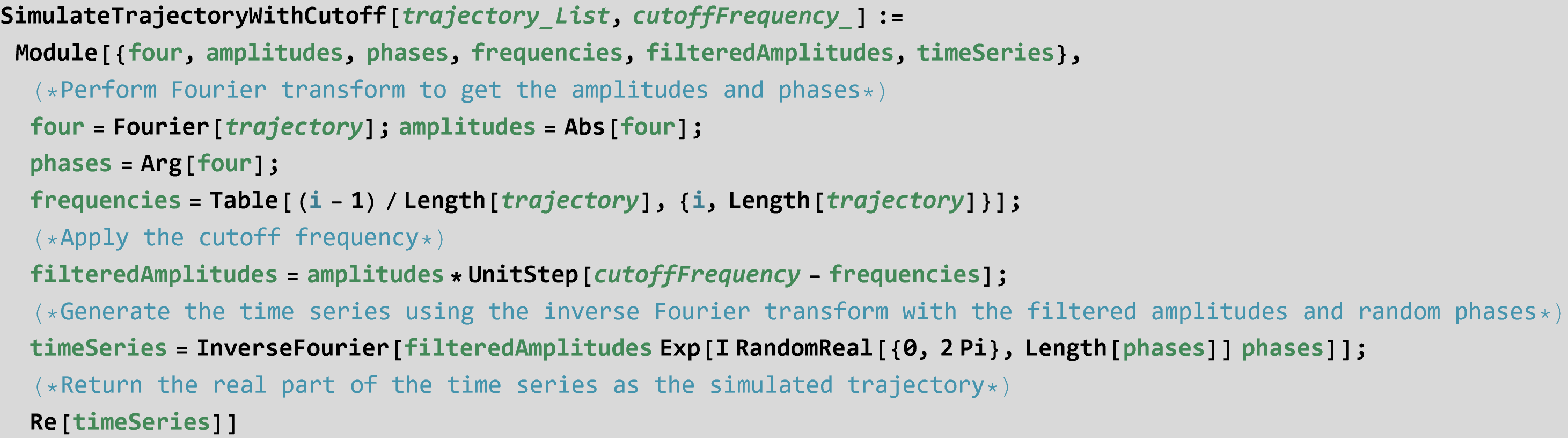


A cut-off parameter served as a high-pass filter to remove high frequencies. This was needed in order to get trajectories that are more similar to the original ones (Supplementary figure 10A-C). We chose to use a cut-off value of 0.05 for the simulated trajectories because it showed the highest resemblance to the real mouse trajectories.


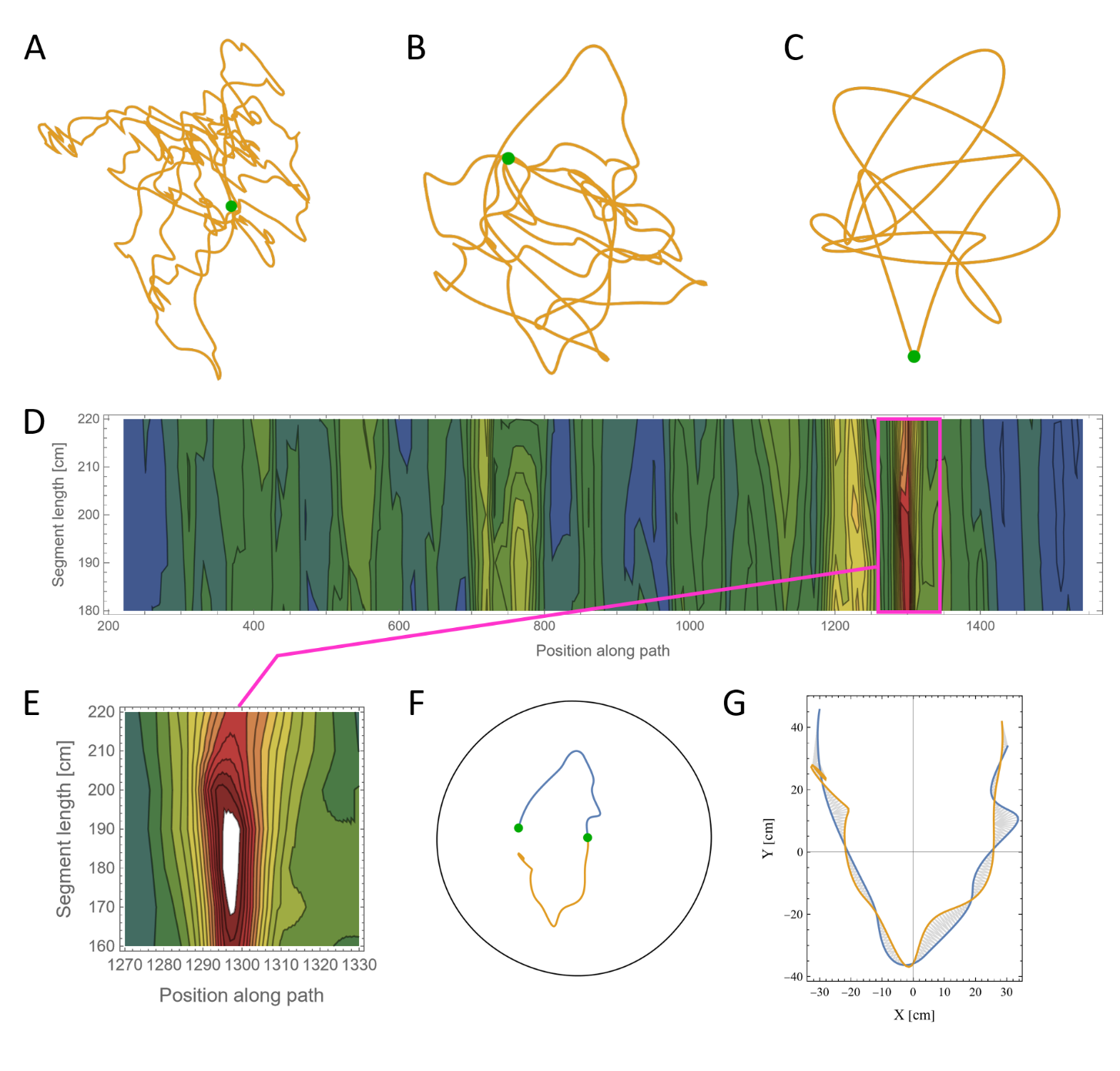


**Supplementary Figure S10.** Simulated trajectories using cut-off values of **(A)** 0.05, **(B)** 0.01, and **(C)** 0.003. Lower values means more high frequencies removed from the spectrum before generating the simulated trajectories, which in turn means smoother trajectories. **(D)** A low-resolution symmetry scan of **(B)**. **(E)** A high-resolution scan of an isolated island of high symmetry (marked in pink). The high-resolution scan included a wider range to ensure finding the peak symmetry value. **(F)** The two successive segments (total length of 360 cm) from the original trajectory (B) that correspond to the peak symmetry value in (E). **(G)** The same segments after centered at the axes' origins and rotated to the best fit (minimal area between lines) demonstrating the high mirror symmetry.

A total of 836m and 796m of arc length were scanned for adjacent and non-adjacent simulated trajectories. The scan included a low resolution scan followed by a high resolution scan of islands of potential high symmetry (see an example in Supplementary figure 10D-G). The analysis was performed in accordance to the selection criteria and scanning procedures described in the methods section. About 7 % of the adjacent- and 8 % out of the non-adjacent- simulated trajectories (in comparison to the 11.1% for the original mouse trajectory) involved long segments showing high symmetry values. Supplementary figure 11 shows both the results of the original comparison (as in Figure 5) and the new scans of the spectral based simulated trajectories. ANOVA test revealed that the symmetry values from the original adjacent segments are also significantly higher than the adjacent simulated trajectories (p-value < 2*10^-8^ (Supplementary table 1).


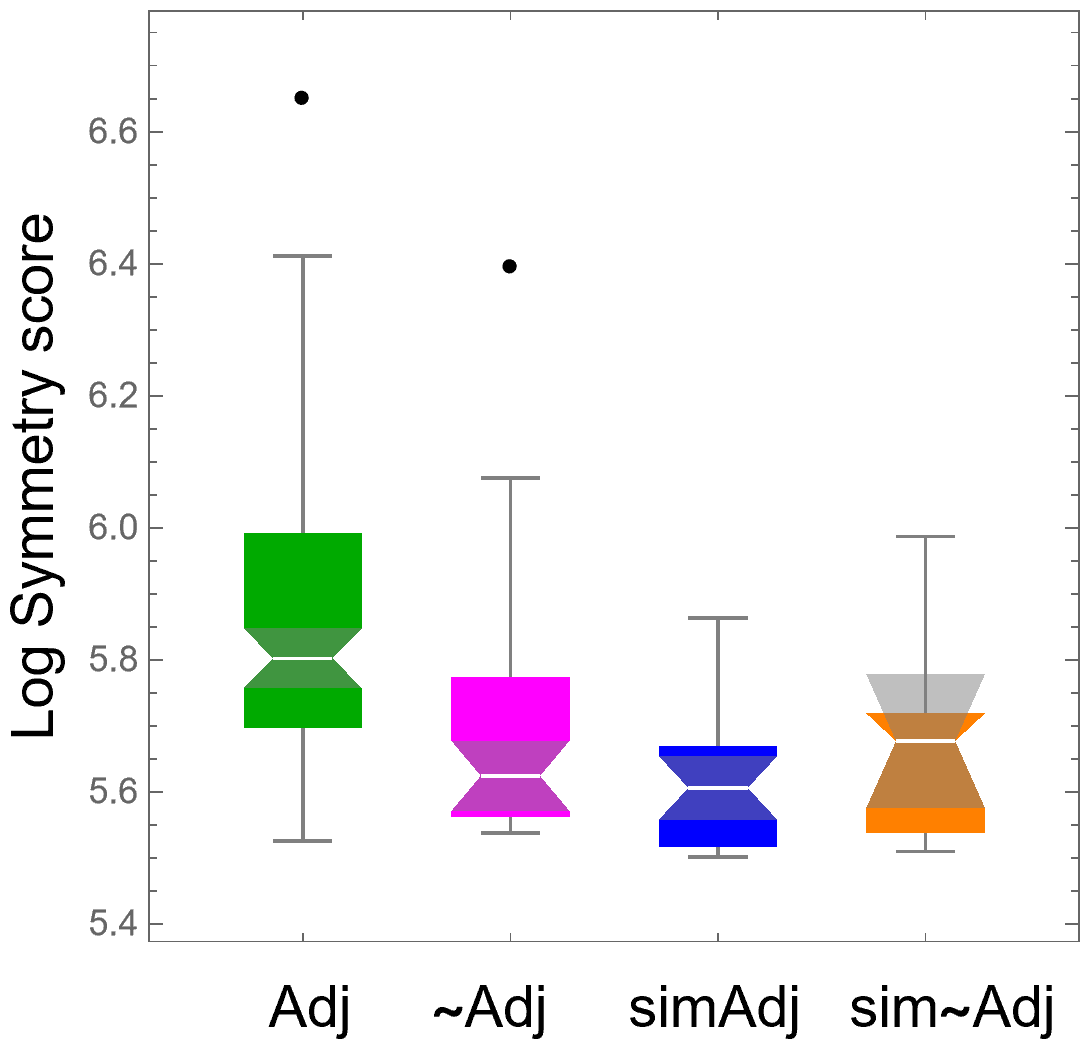


**Supplementary Figure S11.** A box plot representation of the results shown in Figure 6B (green and magenta) with the addition of the results for the simulated trajectories: adjacent segments (in blue) and non-adjacent segments (in orange).


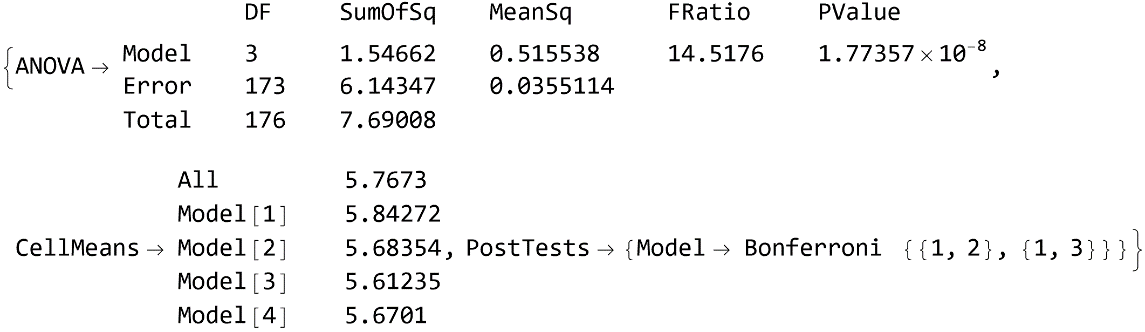


**Supplementary table S1.** The ANOVA results for the comparison between the 4 data sets presented in Supplementary figure 12.
